# Evaluation of pre-hospital transport time of stroke patients to thrombolytic treatment

**DOI:** 10.1186/s13049-014-0065-z

**Published:** 2014-11-13

**Authors:** Sofie Amalie Simonsen, Morten Andresen, Lene Michelsen, Søren Viereck, Freddy K Lippert, Helle Klingenberg Iversen

**Affiliations:** Glostrup Stroke Centre, Department of Neurology, Glostrup Hospital, Copenhagen University Hospital, Nordre ringvej 57, Glostrup, 2600 Denmark; Department of Neurosurgery, Rigshospitalet, Copenhagen University Hospital, Blegdamsvej 9, Copenhagen East, 2100 Denmark; Emergency Medical Services, Copenhagen, Capital Region of Denmark, Telegrafvej 5, Ballerup, 2750 Denmark

**Keywords:** Stroke, Acute cerebral infarction, Pre-hospital delay, Emergency medical services, Logistics, Thrombolysis, Emergency treatment of stroke

## Abstract

**Background:**

Effective treatment of stroke is time dependent. Pre-hospital management is an important link in reducing the time from occurrence of stroke symptoms to effective treatment. The aim of this study was to evaluate time used by emergency medical services (EMS) for stroke patients during a five-year period in order to identify potential delays and evaluate the reorganization of EMS in Copenhagen in 2009.

**Methods:**

We performed a retrospective analysis of ambulance records from stroke patients suitable for thrombolysis from 1 January 2006 to 7 July 2011. We noted response time from dispatch of the ambulance to arrival at the scene, on-scene time and transport time to the hospital—in total, alarm-to-door time. In addition, we noted baseline characteristics.

**Results:**

We reviewed 481 records (58% male, median age 66 years). The median (IQR) alarm-to-door time in minutes was 41 (33–52), of which 18 (12–24) minutes were spent on scene. Response time was reduced from the period before to the period after reorganization (7 vs. 5 minutes, p <0.001). In a linear multiple regression model, higher patient age and longer distance to the hospital correlated with significantly longer transportation time (p <0.001).

**Conclusions:**

This study shows an unchanged alarm-to-door time of 41 minutes over a five-year period. Response time, but not total alarm-to-door time, was reduced during the five years. On-scene time constituted nearly half of the total alarm-to-door time and is thus a point of focus for improvement.

## Background

Every year 12,000 people suffer a stroke in Denmark (population 5.6 million), 15% die during the first month, and at any time, approximately 40,000 citizens are living with disabilities caused by a stroke [1]. Thrombolysis is an emergency treatment known to reduce the damage caused by an ischemic stroke. However, in order to benefit from this treatment, patients must reach the hospital for definitive care within a short time frame; currently this is 4.5 hours for thrombolysis [2]. Early treatment is dependent on several factors; few studies have evaluated these. It is paramount that patients or relatives recognize the symptoms of a stroke, call emergency medical services (EMS) and reach a specialized stroke centre for thrombolysis within the time frame [3-7]. The background for the present study was a general reorganization of EMS in the Capital Region of Denmark in September 2009. In 2009 the medical dispatch was centralized to one dispatch centre managed by the Capital Region, instead of three independent centres run by the individual ambulance providers. Furthermore, specific vehicles were dedicated to emergency ambulance tasks and others to patient transfer. The aim of this study was to evaluate different time intervals spent by EMS for stroke patients during a five-year period including the reorganization in order to identify potential delays.

## Methods

The study is a retrospective review of ambulance records from patients referred to thrombolysis in the period from when thrombolytic treatment was made available at Glostrup Hospital, 1 January 2006, to 7 July 2011. The results are presented for each year as well as in two time intervals, before and after the reorganization of EMS in Copenhagen in September 2009.

In the Capital Region of Denmark, covering 1.7 million inhabitants, there are two centres for thrombolysis for stroke. Potential stroke patients usually call EMS (1-1-2) or their general practitioner, and an ambulance is dispatched as highest priority (lights and sirens). The paramedics evaluate the patient, and if they suspect symptoms or signs of a stroke, the standard operating procedure is to immediately contact the neurologist on call responsible for thrombolysis. By phone the neurologist decides whether the patient is a potential candidate for thrombolytic treatment and thus should be referred directly to the stroke centre. If not, the patient is transferred to the nearest hospital. At the scene or during transportation, the paramedics apply supplementary oxygen, establish two IV accesses and measure vital signs and blood glucose, as well as perform a 12-lead ECG, as standard procedure to prepare for thrombolysis. The patient is assessed for thrombolysis immediately upon arrival at the emergency department.

We noted the time from dispatch of the ambulance to arrival at the scene (response time), the time spent on scene (on-scene time), and the time from departure from the scene to arrival at the stroke centre (transport time). The total time spent was noted as the alarm-to-door time. Furthermore, we noted date, sex, age, postal code where the patient was retrieved, whether the patient was transferred from another hospital and, finally, whether the patient received thrombolytic treatment.

Postal codes were categorized in area groups according to their distance from the stroke centre. Area one is in the radius of 10 kilometres, area two from 11 to 30 kilometres, area three from 31 to 50 kilometres and area four is 51 kilometres or farther from the hospital.

We excluded cases with missing registration of time intervals and special cases where the data was questionable, e.g., when two ambulances were on scene and the reported data was inconsistent.

We included cases even where postal code, sex and age were missing, as the main focus was transport time. Approval from the ethics committee was not required in Denmark for this type of study.

### Statistics

Data analysis was carried out using the statistical software package R. The main results are given in medians and interquartile ranges (IQR). To analyse relationships, we employed linear regression and the Wilcoxon rank-sum test where data was not normally distributed.

## Results

### Patient characteristics

A total of 481 ambulance records were collected. Sixty-nine were excluded: 49 with incomplete documentation of time intervals, 16 with differences in data between two ambulance charts from the same patient, four because stroke was not suspected by EMS. We evaluated 412 ambulance charts, of which 183 were from the first time period and 229 were from the second time period.

Data on postal code was missing in 22 cases, age in five and sex in two cases. These charts were not excluded.

**Figure 1:**
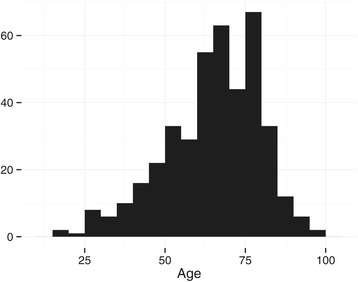
**Age distribution of patients.** Illustrates that the majority of the patients are aged 60 years or above, with a long tail towards the young, where stroke is less common.

**Figure 2:**
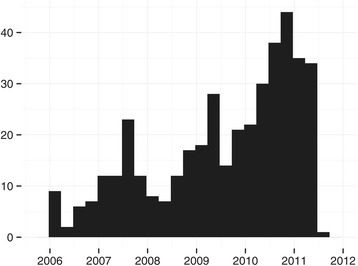
**Number of transports during the study period.** All 412 included transports are shown, both inter-hospital and pre-hospital. We see a sharp rise in patient referrals after January 2010, when inclusion criteria for thrombolysis were expanded [2].

The median age was 66 years (IQR 55–76 years), and 58% of patients were men. The age distribution was not normally distributed and is summarized in Figure 1. Seventy-seven percent of patients received thrombolytic treatment. Number of transports for the duration of the study period was not normally distributed and is illustrated in Figure 2.

For detailed characteristics, the patients were distributed into subgroups according to postal code areas based on distance to the hospital. Seventy-one percent (279) were transported less than 10 kilometres (postal code area one) in order to reach the hospital, and 9% (35) were transported more than 50 kilometres (postal code area four).

### Description of the transport time

The main results are summarized in Table 1. A total of 41 (33–52) minutes were spent from the initial dispatch until arrival at the stroke centre. This constitutes of a response time of 5 (3–8) minutes, on-scene time of 18 (12–24) minutes and transport time of 15 (10–23) minutes. The on-scene time contributed to 44% of the total alarm-to-door time. When reviewing data in the study period split by year, we do not see noteworthy variations. In the year of the reorganization, there does not appear to have been significant changes or negative effects, which could be conceivable following a restructuring effort of this type.

**Table 1:** **Transport time, median (IQR), in subgroups in different time periods and patient groups**

	**Response time**	**On-scene time**	**Transport time**	**Alarm-to-door time**	**N**
All periods	5 (3–8)	18 (12–24)	15 (10–23)	41 (33–52)	412
1 Jan–31 Dec 2006	5 (3–8)	16.5 (11–19)	13.5 (10–17)	35 (28–39)	26
1 Jan–31 Dec 2007	7 (4–10)	18.5 (13–24)	18 (10–32)	44 (35–63)	58
1 Jan–31 Dec 2008	6 (4–9)	16.5 (11–23)	20 (12–29)	44.5 (34–60)	44
1 Jan–31 Dec 2009	7 (4–10)	15.5 (10–21)	15 (11–23)	40 (30–50)	80
1 Jan–31 Dec 2010	5 (3–7)	20 (13–25)	14 (10–22)	42 (34–51)	137
1 Jan–7 Jul 2011	5 (3–6)	18 (12–26)	15 (11–20)	39 (32–50)	67
1 Jan 2006–31 Aug 2009	7 (4–9)	17 (12–23)	16 (10–26)	41 (33–56)	183
1 Sep 2009–7 Jul 2011	5 (3–7)	18 (12–25)	15 (11–21)	41 (33–50)	229
Wilcoxon	p = 0.001*	p = 0.10	p = 0.15	p = 0.39	
Inter-hospital transfer	3 (2–5)	7.5 (5–13)	15 (9–25)	28.5 (22–40)	50
Normal transport	6 (4–9)	19 (14–24)	15 (10–23)	42 (34–53)	362
Wilcoxon	p < 0.0001*	p < 0.0001*	p = 0.72	p < 0.0001*	
Thrombolysis	5 (4–8)	18 (12–23)	15 (10–23)	41 (33–52)	317
No thrombolysis	5 (3–8)	17 (12–24)	16 (11–25)	41 (33–51)	95
Wilcoxon	p = 0.20	p = 0.92	p = 0.50	p = 0.85	

IQR indicates interquartile range, *significant results (p <0.05).

There was a statistically significant reduction in response time between the two periods (7 vs. 5 minutes, p = 0.001), but there were no differences in the on-scene time or the transport time. The two groups are comparable in number, sex and age.

Inter-hospital transfer time for patients having a stroke in-hospital at one of the other hospitals in the region is shorter than the transport time for patients having an out-of-hospital stroke. This is statistically significant regarding response time, on-scene time and alarm-to-door time but not transport time.

No differences in time are seen between the two groups that respectively did and did not undergo thrombolytic treatment.

### Analysis of the transport time

**Figure 3:**
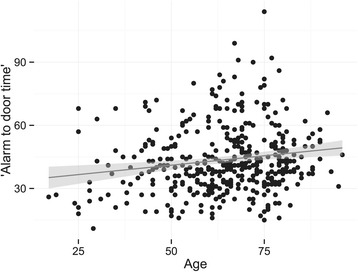
**Scatter plot of alarm-to-door time in minutes by age in years.** The line is the best fit of a linear model, and the grey shadow represents the 95% confidence interval.

**Figure 4:**
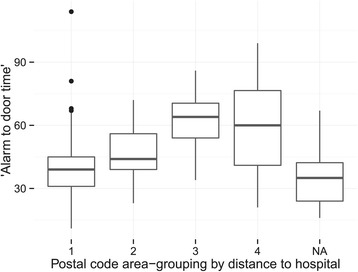
**Box plot of the alarm-to-door time in minutes and postal code areas.** Area 1: <10 km from stroke centre, area 2: 11–30 km from stroke centre, area 3: 31–50 km from stroke centre, area 4: >50 km from stroke centre. NA represents a group where the pick-up location could not be determined. The NA group does not differ significantly from postal area group 1. Note the difference between group 3 and 4 (see Discussion).

We find a significant association between alarm-to-door time and age (p <0.001 and adj. R^2^ = 0.03) and, as expected, alarm-to-door time and postal code area (p <0.001 and adj. R^2^ = 0.26), though only the distance to the hospital really contributes to the variance in alarm-to-door time (summarized in Figures 3 and 4).

We used a linear multiple regression model to describe alarm-to-door time. In this model, 30% of the variation is explained by the parameters sex, age, time of day, season, whether the patient underwent thrombolysis and postal code area. We find a significant association between age (p <0.0001) and postal code area (p <0.0001).

Trends over time show that the response time and transport time were significantly reduced over the course of the study period (p = 0.015, adj. R^2^ = 0.01, and p = 0.03, adj. R^2^ = 0.01 respectively), while on-scene time and alarm-to-door time did not change significantly (p = 0.07 and p = 0.18 respectively). However, the reduction in response time and transport time is too low to have any clinical relevance.

## Discussion

This study shows a total alarm-to-door time of 41 minutes, of which 44% is spent by EMS on scene. It also shows a reduction in response time between the two periods but no difference in total alarm-to-door time. The pre-hospital time intervals and the total time spent from alarm to door are unchanged over a time period of five years, except for response time.

On-scene time contributes to 44% of the total alarm-to-door time and is dependent on several modifiable factors. Reducing the on-scene time, and thereby the total alarm-to-door time, might positively affect the outcome of stroke patients. In our setting, this could be done by providing more detailed information to paramedics about the importance of reduced alarm-to-door time when dealing with stroke patients. Wherever feasible, practical procedures such as IV cannulation, ECG recordings and consultation with the neurologist on call should be done during transport to the hospital, and not on scene. Response time and transport time are satisfyingly low, and because even helicopter transport of stroke patients does not appear to have any effect, these areas are not prime candidates for further intervention [8].

The total time spent when the patient was transferred from another hospital was significantly shorter (7.5 minutes). The reduction in inter-hospital alarm-to-door time is related to the short on-scene time seen when transferring patients from another hospital. The reason for this may be that the preparations have already been done in hospital prior to arrival of the ambulance. If the conference with the neurologist and documentation could be done during the transport, some minutes could probably be saved. The patients in the two groups (inter-hospital transfer and normal transport) are not comparable in number, and the results should be interpreted with caution.

We saw a steady increase in the number of patients evaluated for potential thrombolytic treatment during the study period, though the increase now seems to be levelling off.

With more information available to the public—the potential future patients—about symptoms of stroke and the opportunities for thrombolysis, the number of patients in general may increase.

We demonstrate a significant relationship between the distance to the hospital and the alarm-to-door time. However, this does not apply when the patient was retrieved from over 50 kilometres away from the hospital. Less traffic and quick highway access in rural areas may explain this discrepancy.

Another noteworthy item is the significant association between age and transport time; the older the patient, the longer the alarm-to-door time. However, the literature shows two examples of “the older, the faster” [4,6] while two studies find no association [3,5], and finally, three studies have a similar conclusion to ours, being “the older the patient, the slower the transport” [7,9,10].

Poulakka et al. [11] and Quain et al. [12] assessed the duration from onset of symptoms, instead of the time of initial contact with emergency services, and in the two studies found symptom-to-door times of 71 and 63.5 minutes respectively for patients receiving IV thrombolysis. Morris et al. [3] evaluated all stroke patients regardless of IV thrombolytic treatment and found a mean delay of 2.6 hours from symptoms to arrival, while patients in a study by Maestroni et al. [13] were delayed 5.4 hours. Other studies have included all stroke patients arriving at the emergency department, noting what percentage arrived within, for example, 1, 3 or 6 hours of symptom onset [4-6,9,14]. As the type of data collection and grouping are different, their data is not comparable to ours. However, one study by Mosley et al. [14] of 187 stroke patients from Melbourne, Australia, used the same subgroups as our study. They found a median response time of 12 minutes, an at-scene time of 16 minutes, a transport time of 15 minutes and a total ambulance service time of 44 minutes. Compared to our data, Mosley et al. found a longer response time but a shorter on-scene time. Looking at the total alarm-to-door time, their 44 minutes spent on patient retrieval is almost identical to the 41 minutes that we present in this study. Similarly, Puolakka et al. [11] found an on-scene time of between 18 and 23 minutes. Overall, our results are comparable to earlier studies and indicate that it should be possible to reduce on-scene time.

As stroke treatment is time dependent, the shorter the time interval between symptom onset and final treatment, the better the outcome [15]. Therefore, focus must remain on reducing the time interval from onset of symptoms to treatment. In the pre-hospital setting, this challenge can be addressed in specific areas. Early recognition by patients, bystanders and EMS dispatchers, facilitated by public information campaigns and continuous education, may be part of the solution.

As documented in this study, the on-scene time, which contributes to 44% of the total alarm-to-door time, is an area for further research and intervention.

## Conclusions

This study shows an unchanged alarm-to-door time of 41 minutes over a five-year period, of which 44% (18 minutes) is spent on scene. A reduction in response time between the two periods, but no difference in total alarm-to-door time, is evident. To reduce the time from EMS contact to treatment, and thereby improve the outcome for stroke patients, this study indicates that a reduction in on-scene time should be an area of focus with initiatives including further education of paramedics, as well as optimizing pre-hospital procedures.

## Abbreviation

EMS: Emergency medical services
